# Conceptualizing performance of nursing care as a prerequisite for better measurement: a systematic and interpretive review

**DOI:** 10.1186/1472-6955-12-7

**Published:** 2013-03-07

**Authors:** Carl-Ardy Dubois, Danielle D’Amour, Marie-Pascale Pomey, Francine Girard, Isabelle Brault

**Affiliations:** 1 Faculty of Nursing, University of Montreal, Montreal, Canada; 2 Department of Health Administration, Faculty of Medicine, University of Montreal, Montreal, Canada

**Keywords:** Performance measurement, Nursing care, Systems theory, Quality of care, Nursing structure, Nursing processes, Nursing sensitive outcomes, Systematic review.

## Abstract

**Background:**

Despite the critical role of nursing care in determining high-performing healthcare delivery, performance science in this area is still at an early stage of development and nursing’s contribution most often remains invisible to policy-makers and managers. The objectives of this study were: 1) to develop a theoretically based framework to conceptualize nursing care performance; 2) to analyze how the different components of the framework have been operationalized in the literature; and 3) to develop a pool of indicators sensitive to various aspects of nursing care that can be used as a basis for designing a performance measurement system.

**Methods:**

We carried out a systematic review of published literature across three databases (MEDLINE, EMBASE and CINAHL), focusing on literature between 1990 and 2008. Screening of 2,103 papers resulted in final selection of 101 papers. A detailed template was used to extract the data. For the analysis, we used the method of interpretive synthesis, focusing first on 31 papers with theoretical or conceptual frameworks; the remaining 70 articles were used to strengthen and consolidate the findings.

**Results:**

Current conceptualizations of nursing care performance mostly reflect a system perspective that builds on system theory, Donabedian’s earlier works on healthcare organization, and Parsons’ theory of social action. Drawing on these foundational works and the evidence collated, the Nursing Care Performance Framework (NCPF) we developed conceptualizes nursing care performance as resulting from three nursing subsystems that operate together to achieve three key functions: (1) acquiring, deploying and maintaining nursing resources, (2) transforming nursing resources into nursing services, and (3) producing changes in patients’ conditions. Based on the literature review, these three functions are operationalized through 14 dimensions that cover 51 variables. The NCPF not only specifies core aspects of nursing performance, it also provides decision-makers with a conceptual tool to serve as a common ground from which to define performance, devise a common and balanced set of performance indicators for a given sector of nursing care, and derive benchmarks for this sector.

**Conclusions:**

The NCPF provides a comprehensive, integrated and theoretically based model that allows performance evaluation of both the overall nursing system and its subsystems. Such an approach widens the view of nursing performance to embrace a multidimensional perspective that encompasses the diverse aspects of nursing care.

## Background

Nurses represent the largest occupational group in the healthcare workforce, providing the most care at all levels of the care continuum and accounting for a significant proportion of hospitals’ operating costs [1,2]. Yet nursing’s contribution most often remains virtually invisible to policy-makers and healthcare managers, and many analysts consider it undervalued and understudied [3,4]. Symptomatic of the situation, data considered sensitive to nursing care are, in most cases, not represented in databases that are routinely examined for performance analysis in healthcare organizations and health policy decisions [5,6]. The causes attributed to these symptoms include poor conceptualization of nursing performance, inadequate measures of nursing contribution, inadequate information systems to capture and manipulate nursing performance data, and absence of a standardized language [7,8].

Concerns about the shortage of nurses and its potential adverse effect on patient safety, coupled with mounting public expectations regarding the value of services consumed, have created a sense of urgency about the need for monitoring performance of nursing services [9]. In the current context of health service reforms, driven partly by resource constraints and consumer pressures, increasing demands are being placed on nursing administrators and nursing care providers. They must ensure the performance of nursing services, give a more comprehensive and accurate picture of what they do, and demonstrate the value and benefits of their services in line with established objectives and standards [10,11]. This renewed interest has resulted in an accelerated expansion of a range of initiatives within and outside the nursing profession to make explicit those aspects of care outcomes directly attributable to nursing practice. Specifically, indicators to quantify nursing’s contribution to healthcare performance have been considered in several countries by influential organizations such as the *Agency for Healthcare Research and Quality* and the *National Quality Forum* in the United States, the *Ontario Hospital Association* in Canada, and the *Council on Health Care Standards* in Australia [12,13].

Despite such recent efforts, the research base on performance measurement in the area of nursing care is still considered somewhat embryonic [14], and no common theory-driven schema guides the nursing discipline, the regulatory agencies and the provider organizations in their efforts to define, organize and operationalize the dimensions of nursing care performance [15]. There are many fragmented pieces of knowledge, but what is critically missing is a comprehensive framework in which to fit the pieces together and that can guide the implementation of performance assessment activities in nursing. In many cases, the terminology of performance measurement can itself be confusing and is characterized by a wide array of terms and concepts that include productivity, outcomes, effectiveness, efficiency, quality, etc. As there is no agreed-upon definition, performance measurement systems are also conceptualized in multiple ways [16].

The overall aim of this research was to draw on the most recent developments in conceptual modelling to help build a common orientation on how to capture nursing care performance. More specifically, the objectives of the research were: 1) to develop a theoretically based framework to conceptualize nursing care performance; 2) to analyze how the different components of the framework have been operationalized in the literature; and 3) to develop a pool of indicators sensitive to various aspects of nursing care that can be used as a basis for designing a performance measurement system.

While there is already a substantial amount of literature on the topic of healthcare performance, the focus of our study was on performance metrics potentially sensitive to nursing care. Although other reviews are available in the literature [17,18], none have been as exhaustive and none have systematically analyzed the existing models using a structured template, as we did. Furthermore, while the notions of quality and performance are frequently used interchangeably, our study acknowledges both their conceptual linkages and differences. A system can only be said to be performing if it delivers high quality interventions, care or services [19]. However, performance and quality are not necessarily identical and interchangeable concepts. A widely accepted definition of quality proposed by the Institute of Medicine conceptualizes quality as the degree to which health services for individuals and populations are consistent with current professional knowledge and increase the likelihood of desired health outcomes [20]. From this perspective, quality is at best only a proxy of healthcare performance, which is understood as a much broader concept. To be consistent with developments in contemporary literature, the notion of performance in this study is embedded in a general vision of key functions that an organizational entity must fulfill in a given environment. It refers to the ability of a health unit, organization, or system to perform its diverse functions most effectively and efficiently and to ensure the coordination and equilibrium among these functions needed to achieve its goals. From this perspective, nursing care performance can be measured based on those attributes or dimensions related to the functioning of organizational entities involved in nursing care provision. Those dimensions are not necessarily direct measures of quality, but rather, they cover what an organization does to provide nursing care, how it does it in relation to articulated goals and functions, and the resulting outcomes.

This article presents the Nursing Care Performance Framework (NCPF), describes its key components and identifies indicators related to each component. This NCPF will provide policy-makers and managers with an effective tool to structure performance evaluation systems that are consistent with their overall goals. This framework will widen the view of nursing performance to encompass the diverse aspects of nursing care.

## Methods

**Figure 1:**
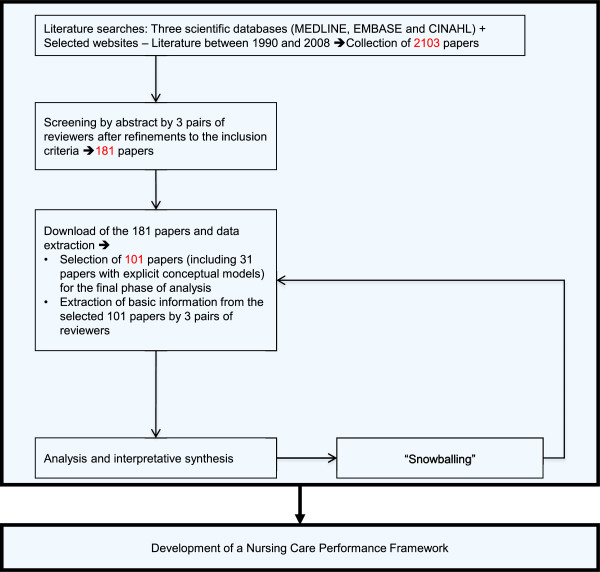
Methodological steps.

To achieve the above-mentioned objectives, our research built upon developments in conceptual modelling and advances in systematic review methods as applied to organizational research [21]. Our approach drew on explicit, systematic and reproducible methodology to locate, select and appraise the available literature and to extract and analyze the relevant data [22,23]. It can be defined as an interpretive synthesis. It is interpretive in the sense that the focus was not on simply aggregating or summarizing data reported in the literature but rather drawing on a large and complex body of evidence to build a conceptual framework grounded in the studies included in the review [24,25]. As summarized in Figure 1, the review followed the main phases that have been systematized in previous works: literature searches, screenings and appraisals, data extraction, and analysis and interpretative synthesis [21,22].

### Literature search

The search for relevant literature was made across three databases. MEDLINE, EMBASE and CINAHL were selected, based on preliminary tests applied to a broader range of databases. Other potential sources, such as the Cochrane and Francis Costello libraries, were rejected after preliminary tests found them unsatisfactory, as they generated hundreds of irrelevant items. After several iterations and advice from a professional librarian, we adopted key words and applied them to the three selected databases. The key words were nursing care performance, nursing performance, nursing-sensitive performance measurement, nursing-sensitive measures, and quality indicators and nursing care. The robustness of the strategy was confirmed by comparison with a predetermined list of key articles referenced in a recent review article [17], all of which were captured by the search when they were clearly related to performance in the area of nursing care. After removal of duplicates, the papers collated at this phase totalled 2,103. The search was limited to literature between 1990 and 2008, to focus on most recent developments in this area. However, reference chaining was used to uncover additional key articles published before this period.

### Screening and appraisal

Our six researchers were paired into three teams and the articles’ abstracts split among the teams. Inclusion criteria were tested on a sample of 15 abstracts. Each abstract was systematically reviewed independently by two researchers based on criteria of: 1) relevance (clear focus on the theme of performance; clear focus on the domain of nursing care); 2) potential contribution to the conceptualization or measurement of performance (development of a theoretical model, elaboration of performance measures, performance evaluation, empirical analysis); and 3) use of explicit and valid methods. For each case, a decision was taken to either exclude the paper or select it for the next step. When there was disagreement, the two researchers met to discuss their views and find common ground. Letters, comments, editorials and anecdotal papers were systematically excluded. The criterion on methodological quality was intentionally broad for this initial screening. At this step, we aimed to include as many relevant papers as possible that met basic methodological standards or were likely to contribute at the level of concepts, while excluding studies that were irrelevant or fatally flawed. One hundred and eighty-one papers passed this screening and advanced to the next step.

### Final screening and data extraction

The 181 papers were downloaded. We designed a data extraction template that was intended, first, to confirm the relevance of each paper (clear focus on performance conceptualization and/or measurement in the domain of nursing care) and its scientific quality (theoretical foundation, design, sampling, data collection, explicit criteria of validity) and then to extract further data. Again, the six researchers were grouped into teams of two. We pretested the data extraction tool on 45 articles split among the three teams, refined the tool, and then applied it to the entire sampling. In-depth examination of each article and data extraction were first completed by one member of a team and then validated by the other. It was still possible at this step to exclude a paper if it was deemed irrelevant or methodologically flawed. Again, when there was disagreement, the two researchers met to discuss their views and find common ground. Of the 181 articles, only 101 merited complete data extraction and analysis.

Information extracted from all the 101 papers included country, type of study, appropriate criteria of validity based on the type of study (ex: representative sampling, data saturation), type of setting, study focus (development of framework, development of indicators, empirical analysis of links between variables) and a list of performance indicators (variables or markers proposed or used to measure performance). The main context of interest was acute care for 65% of the papers, long-term care for 27% and primary care for 8%. This information was supplemented with grey literature, reference chaining and scanning of key journals specializing in performance issues. The three pairs of reviewers were also asked to flag papers with theoretical or conceptual frameworks; 31 such papers were identified on the basis of two criteria: 1) papers with an explicit framework that specified the dimensions of the concept of nursing care performance and/or how these dimensions can be operationalized; and 2) reviews that used a coherently organized structure to present the various aspects of nursing care performance.

### Data analysis

Our approach to analyzing the data involved constructing a general interpretation grounded in the findings of separate studies and then integrating evidence from across studies into a coherent theoretical framework comprising a network of constructs [25,26]. For this phase, the analysis focused first on the 31 papers with theoretical frameworks. Our approach was similar to that often undertaken in primary qualitative research. We began with detailed inspection of the papers, gradually identifying recurring variables used to measure performance. We then grouped these variables into different subdimensions and dimensions, constantly comparing the conceptual structures we were developing against the data in the papers and the models collated, and attempting to specify the relationships between the different variables, dimensions or subdimensions identified. The information extracted from the 70 other papers were incorporated in a second stage to strengthen and consolidate the findings drawn from the first 31 papers, confirm the performance indicators identified and their groupings into dimensions and subdimensions, look for any additional variable or dimension that would not have been captured in the models, and document the relationships between the variables identified. All team members were involved in this analysis and the continual dialogue between them helped to ensure a reflexive account of the processes followed all along the different steps.

## Results and discussion

### A diversity of perspectives with a common thread

A first observation that emerged from the corpus of papers examined was that there are a significant number of activities under way to standardize measures of diverse aspects of nursing work and capture nursing’s contribution to care. The review revealed three parallel streams of activities that have contributed to varying degrees to conceptualizing and measuring performance in nursing care.

A first stream of activities focuses on encoding and classifying all aspects of nursing activities, including nursing diagnoses, nursing interventions and nursing outcomes [27,28]. Those classification systems are viewed as measurable and standardized ways to sort out those health conditions or clinical situations that are amenable to nursing interventions and for which nursing holds the ultimate responsibility for patient outcomes. A second stream of activities, grounded in the patient safety movement, is dominated by a search for evidence relating given factors in nursing structures or processes (e.g. nurse staffing, nursing unit characteristics) to safety failures or poor outcomes for patients [29,30]. A third stream of activities, closely associated with the quality assurance movement, is primarily concerned with developing evidence-based standards of care and measuring resources, processes and interventions that must be in place to optimize care quality and outcomes [31].

While these activities follow different directions, the review also showed they share a common thread. In the three streams, nursing care performance involves the analysis of multiple interacting elements that relate to the diverse aspects of nursing services, their antecedents and their results. This reflects a conception of nursing care as a complex, aggregate entity, comprised of multiple interrelated and interdependent subsystems and components that are logically coordinated and oriented toward the achievement of common goals [32]. As the review unfolded, a core finding that emerged from the analysis was that these diverse streams of activities, to varying extents, built upon Donabedian’s model of healthcare organization and delivery, which in turn owes much to Parsons’ theory of social action and systems theory. Taken together, these theoretical foundations help us understand how inputs are acquired from the nursing care environment and fed into the service production cycle, where transformation of resources results in changes in patient conditions [33]. As such, they provide a strong basis for organizing and integrating the diverse and often fragmented perspectives of nursing care performance.

Donabedian’s structure, processes and outcome (SPO) framework provides a tool that efficiently articulates three subdimensions representing three key components of the healthcare supply chain [34]. In many cases, the three components of the SPO model have been used as a taxonomy to identify and classify those factors or building blocks that must be put together to assess nursing care performance. However, while the SPO triad highlights the different components and the underlying anatomy that define the performance of a given system, it may be not sufficient for understanding the operating mechanisms of these diverse components and their interactions. Parsons’ framework for social analysis provides a functional organismic perspective that affords further insight into the performance of a given system by conceptualizing the social system as a system of interactions between different subsystems, mostly defined in terms of functions [35]. These functions that any system needs to align and balance in order to perform well include goal attainment, production, adaptation to the environment, and values’ maintenance. As such, Parsons’ framework may be useful to understand the physiology of a performance system, conceptualized as a set of specialized functions that must be achieved by autonomous functional subsystems. The Parsons functions provide a generic foundation that has been used to define the criteria on which human services performance must be assessed [36]. This is also congruent with systems theory. A systems approach to nursing care assumes that the nursing system is composed of interrelated subsystems and components that carry out specialized functions. If any of these subsystems performs inadequately, it will affect the performance of the whole system. This means that a highly complex nursing care system must first be broken into subsystems so that each can be analyzed and understood before being reassembled into a whole. Each subsystem must achieve high performance in its own functions but also join with other subsystems and units to optimize the performance of the whole [37]. Although these functional subsystems may be relatively self-sufficient, a systems perspective recognizes the interaction and reciprocal interconnection among their different functions [37].

We used the complementarity of these foundational works to analyze the selected articles and determine the current state of performance measurement in nursing care. Drawing upon these works and the evidence collated, we developed the Nursing Care Performance Framework (NCPF) to integrate diverse perspectives and take into account the diverse aspects of the nursing system. Donabedian’s SPO triad provides the underlying anatomy of this framework. Parsons’ social theory helps to understand the operating mechanisms and functions that underlie each of the constitutive components. The system perspective takes into account the linkages between those different components or subsystems and the environmental factors influencing them. The 31 models we reviewed differ widely in the translation of these theoretical foundations and reveal diverse gaps in measuring nursing care performance. We observed wide differentials in the existing models and approaches regarding how performance is conceptualized and subsequently operationalized. Some models exhibit a plethora of indicators reflecting diverse theoretical, methodological and practical perspectives. Other frameworks focus on selected and restricted aspects of nursing practice, while neglecting others. Structural indicators that may be more easily measurable and more readily available in existing administrative data sets are often put forward to the detriment of more complex process indicators that could better reflect nursing activities. Several frameworks or models reviewed are simply a list of indicators or broad domains and do not clearly indicate the conceptual dimensions and subdimensions that underlie these indicators. While many models built on the Donabedian structure–process–outcomes (SPO) framework, often each component of the triad is taken separately, without attention to the linkages between them. Variations among the existing models also reflect the lack of a common terminology and the use of a plethora of terms and concepts with inconsistent definitions. Despite these limitations, our analysis is built on the hypothesis that conceptualization of performance in the domain of nursing may benefit from the combination of different perspectives and models. Taken together and used in combination, guided by the theoretical foundations mentioned above, existing models may provide a more accurate picture of nursing system performance than would each one separately.

### The nursing care performance framework

**Figure 2:**
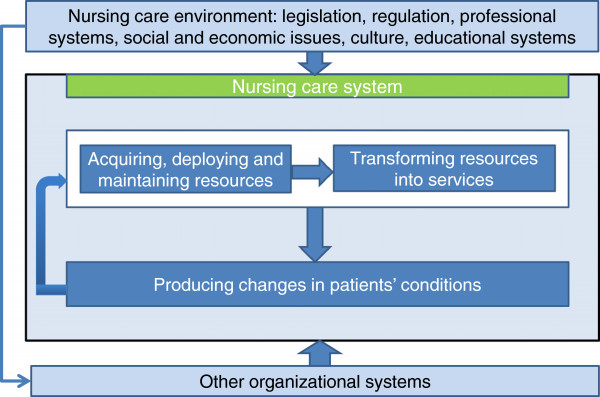
The nursing care performance framework.

The NCPF, illustrated in Figure 2, conceptualizes nursing care performance as resulting from three nursing subsystems that operate together to achieve three key functions: (1) acquiring, deploying and maintaining nursing resources, (2) transforming nursing resources into nursing services, and (3) producing positive changes in a patient’s condition as a result of providing nursing services. These three functions, derived initially from the theoretical foundations mentioned above, served as an analytical tool to capture the main dimensions emerging from efforts to conceptualize and measure performance in the articles reviewed. Table 1 shows how our proposed framework relates to Donabedian’s SPO triad, Parsons’ functional analysis and systems theory and builds on these foundations to develop a broad view of nursing care performance. In the NCPF, each of the three subsystems is composed of multiple components that define the structure, processes and outcomes of the overall system. Within each subsystem, the components are involved in multiple processes of interaction to achieve a specialized function. The underlying systemic perspective highlights the linkages between these functions.

**Table 1:** Theoretical foundations for the NCPF (Nursing Care Performance Framework)

**NCPF three functions**	**Donabedian’s SPO Triad**	**Parsons’ functions**	**Systems theory**
Acquiring, deploying and maintaining nursing resources	**Structure:** refers to characteristics that affect the ability of the nursing system to meet healthcare needs	**Adaptation:** relates to a nursing system’s capacity to acquire and maintain the resources it needs, develop new resources or improve allocation of its resources	Ability of the nursing system, as an open system composed of interrelated subsystems, to acquire inputs from its environment, engage in transformation processes and generate output that brings about an added value for its environment
Transforming nursing resources into nursing services	**Process:** refers to the nature of activities done by nurses in providing care and the characteristics of the practice environment	**Production:** relates to the ability of the nursing system to coordinate the efforts of its constituencies and ensure smooth functioning of processes involved in providing nursing services	
		**Value maintenance**: relates to creating and maintaining values and standards that guide choices in the design of nursing services	
Producing changes in a patient’s condition as a result of providing nursing services	**Outcome:** refers to states of health or events that follow nursing care and are affected by nursing care	**Goal attainment:** relates to a nursing system’s capacity to fulfill its mission and bring about a valued state in the system’s relationships to its environments	

Building upon the perspectives mentioned above, the NCPF suggests a broader view of nursing care performance that can be defined as the capacity demonstrated by an organization or an organizational unit to acquire the needed nursing resources and use them in a sustainable manner to produce nursing services that effectively improve patients’ conditions. Contrary to many prevailing definitions, nursing care performance is not restricted to the end goals or outcomes of the nursing system, but refers also to the effectiveness of those upstream functions that provide the means necessary to achieve nursing system goals.

As shown in Tables 2, 3 and 4, the three functions of the NCPF summarize the universe of dimensions and subdimensions used to conceptualize nursing care performance in the 31 models collated (see the models list, Table 5). The same dimensions and subdimensions were found in the 70 additional articles (empirical analyses, descriptive articles) retained after the screening exercise. Tables 2, 3 and 4 show not only the extent to which the 31 performance models inventoried integrate the three subsystems, but also how each subsystem is operationalized in each model. Assuming these models summarize the universe of content commonly accepted as the main components of nursing care performance, we listed all variables present in the 31 models. Variables with similar meanings but differences in denominations were renamed to facilitate their groupings under one heading. We present below the three nursing care subsystems and their components. We also provide evidence on interrelations both between each subsystem’s components and between the subsystems themselves.

**Table 2:** Operationalization of the subsystem of acquiring, deploying and maintaining nursing resources

**Acquiring, deploying and maintaining nursing resources**	**1**	**2**	**3**	**4**	**5**	**6**	**7**	**8**	**9**	**10**	**11**	**12**	**13**	**14**	**15**	**16**	**17**	**18**	**19**	**20**	**21**	**22**	**23**	**24**	**25**	**26**	**27**	**28**	**29**	**30**	**31**	**# models**
**1. Nursing staff supply**																																
*Quantity/intensity*	1				1	1		1		1	1	1		1		1			1			1		1	1	1			1	1	1	**17**
*Quality (training, experience)*		1			1		1	1		1	1	1		1		1		1	1			1	1		1	1	1			1	1	**18**
*Skill mix*	1				1	1		1		1	1			1		1		1	1			1	1	1	1	1			1	1	1	**18**
*Patient classification systems*	1				1	1	1		1		1	1		1		1			1							1		1				**12**
**2. Working conditions**																																
**a. Support resources**																																
*Physical facilities*	1				1	1				1		1				1		1														**7**
*Material resources*	1					1																				1						**3**
**b. Employment conditions**																																
*Stability (Overtime/ agency nurses floating nurses)*																1						1	1		1						1	**5**
*Workload*	1						1							1					1			1	1			1	1					**8**
**3. Staff maintenance**																																
*Satisfaction at work*	1					1		1				1		1								1	1	1							1	**9**
*Work-related accidents, injuries, illnesses*																							1			1						**2**
*Retention/turnover*	1							1		1		1											1		1				1	1	1	**9**
*Absenteeism*	1							1				1				1							1		1							**6**
**4. Economic sustainability**																																
*Cost of resources*	1											1															1					**3**
*Cost per case-mix or patient-day*	1			1			1	1		1		1						1		1			1		1	1						**11**
**Number of indicators**	**11**	**1**	**0**	**1**	**5**	**6**	**4**	**7**	**1**	**6**	**4**	**9**	**0**	**6**	**0**	**7**	**0**	**4**	**5**	**1**	**0**	**6**	**9**	**3**	**7**	**8**	**3**	**1**	**3**	**4**	**6**	

**Table 3:** Operationalization of the subsystem of transforming nursing resources into relevant nursing services

**Transforming nursing resources into relevant nursing services**	**1**	**2**	**3**	**4**	**5**	**6**	**7**	**8**	**9**	**10**	**11**	**12**	**13**	**14**	**15**	**16**	**17**	**18**	**19**	**20**	**21**	**22**	**23**	**24**	**25**	**26**	**27**	**28**	**29**	**30**	**31**	**# models**
**1. Nursing processes**																																
*Assessment, planning & evaluation*				1				1	1	1	1	1	1												1			1				**9**
*Problems & symptoms management*				1		1		1	1	1					1	1			1	1			1		1		1	1				**13**
*Promotion / Prevention*						1							1						1	1			1	1	1							**7**
*Hospital community integration/ Discharge planning*													1			1				1						1						**4**
*Deployment of scope of practice*												1		1								1										**3**
**2. Patient centrality in the nursing care delivery process**																																
*Continuity (reactivity, timeliness, coordination)*		1		1			1					1				1		1	1				1									**8**
*Patient/family involvement (self-care/ information/ education)*			1		1	1	1			1		1	1					1	1				1		1	1	1					**13**
*Responsiveness to patients’ needs and expectations (communication, comprehensiveness)*					1		1					1											1									**4**
**3. Nursing work environment**																																
*Nursing work environment characteristics (perceived autonomy, role tension, collaboration)*		1					1				1	1		1	1				1			1			1	1			1		1	**12**
**4. Professional satisfaction**	1					1						1		1																	1	**5**
**Number of indicators**	**2**	**2**	**1**	**3**	**2**	**5**	**4**	**2**	**2**	**3**	**2**	**7**	**4**	**3**	**2**	**3**	**0**	**2**	**5**	**3**	**0**	**2**	**5**	**1**	**5**	**3**	**2**	**2**	**1**	**0**	**3**	

**Table 4:** Operationalization of the subsystem of producing changes in patients’ condition

**Producing changes in patients’ conditions**	**1**	**2**	**3**	**4**	**5**	**6**	**7**	**8**	**9**	**10**	**11**	**12**	**13**	**14**	**15**	**16**	**17**	**18**	**19**	**20**	**21**	**22**	**23**	**24**	**25**	**26**	**27**	**28**	**29**	**30**	**31**	**# models**
**1. Risk outcomes and safety**																																
*Falls*	1		1			1		1		1		1	1	1									1	1	1	1	1	1	1	1	1	**17**
*Injuries*										1			1											1					1	1	1	**6**
*Medication management: errors and complications*	1		1					1		1		1	1	1	1							1			1	1	1	1		1		**14**
*Pulmonary infections*													1	1								1			1				1			**5**
*Pressure ulcers/skin integrity*			1			1		1		1		1	1	1	1	1	1					1	1	1	1	°			1	1	1	**17**
*Urinary complications*										1			1	1		1									1				1			**6**
*Intravenous infections*						1																1	1	1	1				1	1		**7**
*Abuses*										1			1																		1	**3**
*Nosocomial infections (taken broadly)*	1		1	1				1				1		1			1					1		1	1	1					1	**12**
*Failure to rescue*																1							1		1	1						**4**
**2. Patient comfort and quality of life related to care**																																
*Hygiene*			1					1		1					1																	**4**
*Physical and chemical restraints*						1				1	1		1										1		1				1	1		**8**
*Symptoms management (*e.g.*, pain, nausea, dyspnoea, fever)*	1		1	1	1	1	1	1				1	1		1	1	1	1	1	1	1		1	1	1		1			1	1	**22**
*Incontinence*			1							1			1		1		1															**5**
*Comfort and quality of life (taken broadly)*																				1			1				1					**3**
**3. Patient empowerment**																																
*Ability to achieve appropriate self-care*		1		1		1						1		1		1	1	1	1	1	1											**11**
*Adoption of health-promoting behaviours*																		1			1											**2**
**4. Patient functional status**																																
*Physical functional capacity*		1		1	1	1	1	1		1		1				1	1	1	1	1	1		1		1		1					**17**
*Cognitive and psychosocial functional capacity*		1		1	1	1	1	1		1		1				1	1	1		1	1		1				1					**15**
*Functional capacity (taken broadly)*		1		1			1																									**3**
*Recovery of initial health status*									1													1					1					**3**
*Nutritional status*			1					1		1			1				1				1											**6**
**5. Patient satisfaction**																																
*Patient satisfaction/complaints*	1	1	1	1	1	1	1	1				1		1		1		1				1	1	1	1		1					**17**
***6. *Joint contribution of nursing with other care systems**																																
*Hospital mortality*								1	1			1										1			1	1	1	1	1			**9**
*Readmissions*			1	1								1								1					1		1					**6**
*Length of stay*	1			1			1	1				1								1		1			1		1					**9**
*Other complications related to care interventions*				1			1					1								1		1					1		1			**7**
**Number of outcomes indicators**	**6**	**5**	**10**	**10**	**4**	**9**	**7**	**12**	**2**	**12**	**1**	**13**	**11**	**8**	**5**	**8**	**8**	**6**	**3**	**8**	**6**	**10**	**10**	**7**	**15**	**5**	**12**	**3**	**9**	**7**	**6**	

**Table 5:** Models list

**# paper**	**Model**	**References**
1	Conceptualization of nursing service organization as a system	[10]
2	Nursing role effectiveness model	[38]
3	Approaches for selecting and developing indicators	[18]
4	Nursing role effectiveness model - Application to quality improvement	[39]
5	Framework for quality of care with cell characteristics	[40]
6	The health care vortex: acute care quality indicators; community-based non-acute care indicators	[41]
7	Conceptual framework for evaluating the ACNP role (adapted from the Nursing Role Effectiveness Model)	[42]
8	SPO framework	[43]
9	Process of nursing care ratings	[44]
10	SPO systems model of nursing care quality in nursing homes	[45]
11	Adapted framework from the Quality Health Outcomes Model (Mitchell et al., 1998)	[46]
12	Antecedents or structural criteria of quality nursing care - Process criteria of quality nursing care - Consequences or outcome criteria of quality nursing care	[47]
13	Patient safety related outcomes and adverse events per category of preventive nursing activity	[48]
14	Conceptual framework for the RICH nursing study (Rationing of Nursing Care in Switzerland study)	[49]
15	Expert panel concept model	[50]
16	Ontario Hospital Report framework - Indicators in the nursing report	[51,52]
17	Nursing Outcomes Classification	[53]
18	A theory driven approach to evaluating quality of nursing care	[54]
19	Nursing role effectiveness model	[55]
20	Oncology Nursing Society Outcomes project team	[56]
21	Nursing sensitive patient outcomes (NOC)	[57]
22	Conceptual framework of ICU nursing workload	[58]
23	The Spider diagram nursing quality report card	[59]
24	ANA acute care nursing quality indicators	[60]
25	Review - Summary of conceptual frameworks for measurement	[17]
26	Nurse staffing and patient outcomes model	[61]
27	A classification scheme for outcomes indicators	[62]
28	Components of quality rating	[63]
29	National voluntary consensus standards for nursing sensitive care	[64]
30	CalNOC variables for the virtual dashboard	[65]
31	Nursing sensitive indicators	[66]

### Acquiring, deploying and maintaining nursing resources

No system for healthcare delivery can fulfill its objective of providing care and improving patient health without deploying the necessary human and material resources. Therefore, as revealed by our review of the 31 models, the first key function of a nursing system is to acquire, deploy and maintain the resources needed to provide nursing care. Twenty-six of the models include indicators related to this function, which has been defined as the ability to ensure a sound and efficient stewardship in acquiring and managing the needed nursing resources [67]. To achieve this function, the nursing system must perform on four key dimensions (see Table 2).

#### Supply of nursing staff

Nursing care is labour-intensive, and delivering effective nursing services depends on the availability of staff with the skills and competencies necessary to address patients’ specific needs in a timely manner. The supply of nursing staff, as a performance measure, thus reflects the effectiveness of diverse activities that govern nursing staff intake (planning, recruitment, selection) and deployment and must ensure an adequate balance with the demand for nursing services. Supply is concerned with not only the quantity of available staff, but also their types (educational preparation, qualifications, and experience) and their mix. It must also be examined in the capacity of available staff to address the specific needs of patients they care for, and this raises the importance of a resource allocation system that takes into account the acuity of patients’ conditions.

#### Nurses’ working conditions

Two aspects of this dimension emerged from the literature reviewed. One was the diverse types of material resources that are required to support the work of nursing staff. These include physical facilities, technologies, organizational configurations and financial resources, all factors identified in several models as precursors that define the overall organization of nursing care and determine the extent to which nursing staff are able to perform their roles. The other aspect consisted of employment characteristics, which encompass a number of issues ranging from workload, scheduling and employment status to system-wide issues such as labour relationships. This aspect reflects the ability to create conditions that may attract nursing workers and ensure their stability in the workforce. This aspect is captured in the inventoried models by a series of indicators, including balance between temporary and permanent staff, use of agency personnel, ratios of floating staff, balance between full-time and part-time staff, and ratios of overtime hours.

#### Maintenance and economic sustainability of the nursing staff

These two dimensions can be considered to a certain extent as contingent upon activities developed in the two first dimensions (supply and working conditions). Poor working conditions and inadequate staffing, often reflected in heavy workload, unpredictable and inflexible schedules, excessive overtime, and threat of job loss, influence workforce maintenance by limiting the capacity to recruit new nurses and retain those already employed. It has been demonstrated that the same factors are significant predictors of work-related illnesses, injuries and job dissatisfaction, which in turn lead to increased absenteeism and turnover [68-71]. The costs associated with these adverse effects come in diverse forms: productivity costs, wage replacements and disability payouts.

Beyond the ability to manage the inevitable outflow of resources, a perennial challenge for every organization is to sustain its economic capacity to acquire and maintain the resources needed. This sustainability reflects a unit’s or organization’s ability to obtain needed inputs as economically as possible (that is, obtaining appropriate quality resources at least cost). Other indicators, such as cost per case-mix, highlight the issue of productivity and the imperative of maximizing the outputs produced from a given set of inputs or, alternatively, minimizing the inputs of nursing tasks, materials, and equipment to produce nursing services.

Thus, with regard to this first function, nursing system performance covers a set of four dimensions that were operationalized into 14 variables. Five of the 31 models have no clear variables related to this subsystem. In the remaining 26, the number of variables used to conceptualize this subsystem varies widely, from one to 11. The most prevalent variables were related to nursing staff supply: quantity/intensity (17 times), quality (18) and skill mix (18). The issue of cost per case-mix figures in 11 models.

The usefulness of indicators related to this first subsystem for assessing nursing care performance may be justified on several grounds:

#### Their potential to detect latent failures at the “blunt end” of the nursing system

The indicators mentioned above may reveal a broad set of dysfunctions and deficiencies, such as insufficient supply of basic material resources, gaps between nurse staffing and patients’ needs, heavy workload and staff instability. Such deficiencies are considered as latent failures that do not involve the practitioner and do not result from direct contact with the patients but that may have potentially delayed consequences on both nursing care processes and outcomes. As an example, inappropriate nurse staffing may not necessary result in immediate harm for patients but reveals a latent failure that may force nurses to reduce the surveillance function, thus creating unsafe delivery conditions that may ultimately result in adverse events among patients.

#### Their sensitivity to nursing management

These indicators’ sensitivity to nursing management (e.g. staffing decisions) gives them an explanatory value for differing performances across care units or organizations. The four dimensions that define this subsystem are all modifiable factors that result from managerial decisions and can be enhanced through policy initiatives at the unit, organizational and system levels.

#### Their linkage with nursing care processes

Assessing this function makes it possible to judge whether nursing care is being provided under conditions that are conducive to optimal nursing processes. Although the link between structural attributes and care processes has been shown to be inconsistent, the literature reviewed contains a number of examples illustrating the potential application of structural indicators to predicting the performance of nursing processes. Research has shown that nursing inputs influence RNs’ ability to care for patients, therefore affecting surveillance and other processes of care [72]. Higher patient-to-RN ratios and nursing workload have been associated with more tasks left undone by RNs and rationing of nursing care [49,73]. Low levels of staffing or heavy workload may result in reductions in time spent by nurses collaborating and communicating with other providers and patients, thereby affecting the quality of both interprofessional coordination [74] and nurse-patient communication [75].

#### Their linkage with patient outcomes

Measures of such structural aspects may be also useful to predict patient outcomes or to specify the conditions under which outcomes are produced. A number of studies and systematic reviews suggest that higher staffing levels and a richer staff-mix and skill-mix may be associated with better outcomes and fewer adverse events for patients [76,77]. Conversely, poor working conditions, inadequate staffing resulting in heavy workload or extended working hours, and problems reflective of staff maintenance, such as high turnover and excessive utilization of temporary staff from external agencies, have been linked with increasing risks to patient safety [78-81].

### Transforming nursing resources into relevant nursing services

The effective stewardship of nursing resources, as described above, is not an end state. A second key function of the nursing system is to transform available resources into nursing services that address patients’ needs. This function involves a broad set of processes and mechanisms that reflect not only what staff nurses do for, with, and on behalf of patients and their families, but also what nursing managers do to support nurses’ work and create an appropriate practice environment. It encompasses, as well, how patients are engaged in their own care processes and how both staff and patients live their experience of care. Among the 31 models inventoried, 28 include indicators related to this function. Those are covered in four interrelated dimensions, described below.

#### The nursing processes

Nursing care provision involves a range of individual, family, community and population-based approaches. It is materialized through interventions and processes that reflect the deployment of nurses’ scope of practice, including assessment, planning and evaluation, problem and symptom management, health promotion and illness prevention, care coordination, and discharge planning. From a provider perspective, they capture the technical aspects of care and reflect the degree to which staff are able to use all their competencies and deploy their full scope of practice. Such processes also reflect the ability to provide care that meets the patient’s overall needs.

#### Nurses’ practice environments

While effective nursing interventions are considered antecedents of health outcomes, nurses’ ability to perform these interventions is closely and consistently associated with organizational processes that define the nursing practice environment and mediate the outcomes [54,82]. These processes have been conceptualized as interventions to support nursing work and create a professional environment for nurses [83]. Many of the indicators for capturing these processes that emerged from this review echo the characteristics of magnet organizations. These indicators include: support for nurses from co-workers and administration; communication and collaboration among nursing staff; collegiality between nurses and other professional groups; a motivating work climate; decentralization of decision-making with responsibilities for nursing services devolved to the nursing unit; and autonomy for nurses in their role.

#### Patient experience and professional satisfaction

These two dimensions are to a certain extent contingent upon the first two. Patient experience can be considered the result of clinical and organizational processes that should optimally ensure patients receive the right nursing care, at the right time, and in the right way for them. Such a measure is essential to assess the acceptability and appropriateness of nursing care from the patient perspective. A number of frameworks have been developed to conceptualize patient experience [20,84-87]. Yet the central theme, overarching all others in the nursing literature reviewed, relates to the patient-centredness of nursing care. Holistic, patient-centred care has often been seen as the essence of nursing care and interpreted as nurses’ ability to communicate effectively with patients, work with patients’ expectations and values, engage patients in their care, deliver services that are sensitive to patients’ needs, and ensure patients’ smooth transition across the care system [88,89]. This notion covers not only the technical and organizational aspects of nursing processes, but also their interpersonal dimension. It is reflected in the 31 models through a number of indicators that we grouped inductively into three attributes: 1) care continuity, which includes diverse facets, such as coordination of care, patient transition, and intra- and interprofessional collaboration; 2) patient/family involvement, which is concerned with engaging patients and their families in their care, and the types of support provided to them to empower them and foster self-care; and 3) responsiveness, which also encompasses several facets, including respect for patients’ preferences, quality of communication with patients and their families and degree of comprehensiveness of care.

Nurses’ professional satisfaction may be also considered the result of nursing processes. Although nurses can be rewarded through different facets of their work, their professional satisfaction mostly results from specific aspects that influence their perception of their ability to accomplish their daily assignments and enjoy the work itself. As such, professional satisfaction relates to those Herzberg motivator factors and intrinsic rewards that are directly linked with the work itself (e.g. responsibility, achievement, growth) as opposed to hygiene factors (e.g. salary, working conditions, supervision) [90]. Measures of professional satisfaction have also built upon the work of Hinshaw and colleagues, for whom nurses’ job satisfaction is a combined function of their perception of the quality of care they provide, having the time to do their job, and the enjoyment derived from it [91].

Table 3 summarizes how this second subsystem is conceptualized and operationalized within the 31 conceptual models reviewed. In total, the review identified 10 variables split across the four dimensions. Only three models have no indicator related to this subsystem. For the remaining 28, the number of variables used to conceptualize this subsystem ranges from one to seven. The most prevalent variables in these models are patient/family involvement (13 times); problems and symptoms management (13), nursing work practice environment characteristics (12), assessment, planning and evaluation (8) and care continuity (8).

The usefulness of these four sets of indicators may be justified on three main grounds:

#### Their potential to detect active or imminent failures at the “sharp end” of the nursing system

An intrinsic advantage of indicators related to this function is that they are more sensitive to differences in quality of care than are structure and outcome indicators. They may reveal dysfunctions and deficiencies in the clinical microsystem, where providers interact directly with patients to deliver care. Inappropriate nurse interventions, miscommunication between nursing team members, and continuity gaps are examples of dysfunctions that create holes in the nursing system’s front line of defense. Such holes are immediate causes of imminent or active failures that undermine the patient’s experience of nursing care, compromise the provision of adequate services, predispose those services to errors, and ultimately may result in adverse outcomes.

#### Their sensitivity to nurse interventions

These indicators refer to the essence of what nurses do to improve patients’ conditions [92]. They provide insights into the clinical and organizational processes that reflect both how nurses enact their roles and the conditions experienced by patients in their interactions with the nursing system. They also provide information that is actionable. Knowing which processes are ineffective is essential for both readjusting how nursing care is provided and redeploying nursing resources.

#### Their linkage with patient outcomes

Specific nursing processes, characterized by patient involvement, patient education, self-care assistance and psychological support, have been associated with better outcomes for patients [93-95]. Doran and colleagues provided evidence that patients’ therapeutic self-care ability was predicted by nurses’ coordination of care, communication with other health care providers, and provision of high-quality technical care [38]. Other studies have shown that organizational models of nursing care which result in greater nurse autonomy, more control by nurses of resources, and better relations between nurses and physicians yield better patient satisfaction and outcomes [96,97].

### Producing positive changes in patients’ conditions

Beyond the imperatives of effective stewardship of nursing resources and their transformation into services that meet high standards of quality, the desired end result of the interactions between patients, nursing staff, and nursing processes is to produce outcomes that lead to positive changes in a patient’s functional status, disease state or evolving condition. All 31 models include indicators related to this third function. Using an inductive process, we identified six distinct but interrelated dimensions from the plethora of outcome indicators in the literature reviewed. The first five categories are considered nursing-sensitive outcomes, that is, outcomes that are affected, provided, and/or influenced by nursing personnel [80,98], although nursing may not be exclusively responsible for them. The sixth category includes outcomes to which nurses may contribute to some extent, but which are less informative for assessing nursing interventions because these outcomes may be more influenced by other systems.

#### Outcomes reflecting patient safety

Nursing care services are provided to patients in an environment with complex interactions that can generate harm, errors and unintended outcomes. Because of this, patient safety is largely considered an indicator of high-performing nursing care. This is related to nurses’ roles in integrating care, detecting hazards and, ultimately, preventing errors and adverse events [99,100]. Failures in fulfilling these roles may result in errors of omission and commission, as well as adverse events. This review identified a number of safety-related outcomes considered potentially sensitive to nursing: patient falls, injuries, medication errors, pulmonary infections, pressure ulcers, urinary tract infections, intravenous infections, abuses, and failure to rescue.

#### Outcomes reflecting patient comfort and quality of life related to care

Beyond harm prevention, the value of nursing care services lies also in their potential to relieve discomfort and improve the quality of life in the context of care. From this standpoint, nursing system performance reflects the extent to which patients’ needs in relation to personal hygiene, nutrition, management of symptoms (pain, dyspnea), and continence are met, unnecessary interventions (physical or chemical restraints, nasogastric tubes, prolonged use of urinary catheters) are avoided, and patients’ respect is ensured throughout the episode of care.

#### Outcomes reflecting changes in patients’ knowledge, skills, and behaviours

Such outcomes reflect patients’ ability to influence their own conditions through self-care and new behaviours. The indicators here focus on knowledge gained, attitudes changed, skills acquired, and behaviours adopted as a result of nursing interventions. Examples include patients’ knowledge of the prescribed treatment, ability to recognize symptoms, ability to carry out treatments as prescribed, and demonstration of health promoting behaviours.

#### Outcomes reflecting patients’ functional status

This category of indicators covers essential end results and benefits that reflect what happens in people’s lives as a result of nursing care interventions. In the models examined, these indicators encompass diverse aspects of patients’ general functional status and conditions, including physical, psychosocial and cognitive status.

#### Outcomes reflecting patient satisfaction with the care experience

These indicators focus on patients’ satisfaction with their care experience and the evolution of their condition. This subjective evaluation by patients reflects the interaction of their expectations of care and their perceptions of actual outcomes resulting from provider services [101]. The nature of nurses’ responsibilities is such that they are potentially well positioned to influence patients’ conditions and how the health system functions across all aspects of patient care. Hence, patients’ satisfaction with nursing care is considered in several models to be both an outcome of nursing services and a primary determinant of overall satisfaction with an episode of care.

#### Outcomes reflecting a joint contribution of nursing care and other care systems

Nursing as a “whole system” interacts with several other “whole systems” to achieve outcomes. Positive change in patients’ health status is an ultimate outcome that most often reflects the contribution of several systems of care and many healthcare disciplines. Conversely, negative outcomes such as mortality, morbidity, complications, extended length of stay and readmissions reflect failures that may have multiple causes and can originate from one or more systems. Although such events cannot easily be imputed either to nursing or to any specific care system, they still reflect outcomes of each of these systems and warrant consideration in assessing the performance of any system.

Table 4 shows how this third function is conceptualized and operationalized in the models reviewed. In total, the review identified 27 variables, which we grouped into six dimensions. All 31 models include outcome indicators. The number of variables used to conceptualize this subsystem ranges from one to 15. The most prevalent variables cut across the different categories of nursing-sensitive outcomes and include symptom management (22 times), skin integrity (17), falls (17), patient satisfaction (17), physical functional capacity (17), cognitive and psychosocial functional capacity (15), medication management (14), nosocomial infections (12) and ability to achieve appropriate self-care (11). The linkages between these outcomes related indicators and the two other subsystems have been described in the previous sections.

## Conclusions

**Figure 3:**
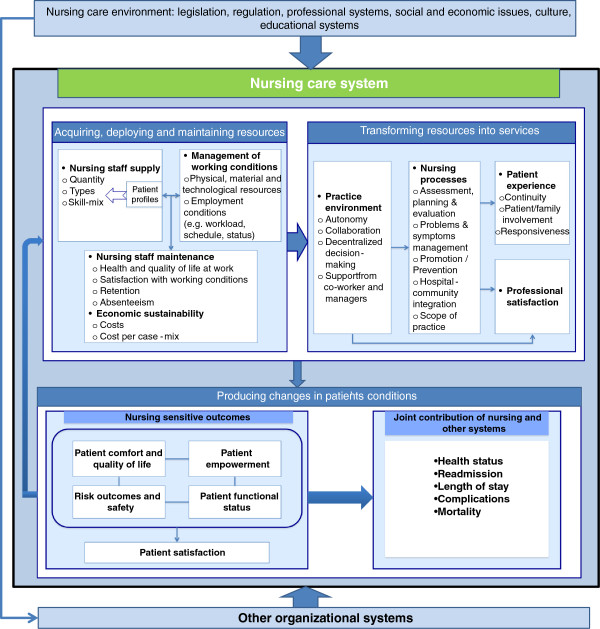
The nursing care performance framework.

Across the three subsystems, at least 51 variables, grouped into 14 dimensions, have been used to assess these subsystems’ performance in achieving their functions. Cross-validation by examination of the 70 additional papers revealed no additional generic variables, but in some cases, specific indicators related to a specific clinical domain and linkages between those variables. Figure 3 summarizes the dimensions and indicators inventoried for the three subsystems, based on the review.

While all 51 variables are valid indicators that together represent the entire domain of nursing care performance as a construct, it can be seen in Tables 2, 3 and 4 that no individual model covers this entire domain. Rather, the different models show a variety of combinations of potential variables, reflecting the diversity of options for measuring nursing care. However, it is worth noting that, for nearly all the models, the scope of nursing care performance variables extends to more than one subsystem. In fact, 25 of the 31 models include indicators related to all three subsystems. Of the others, three focus on the second and third subsystems, one includes indicators related to the first and third subsystems, and two restrict their scope to outcome indicators.

From a policy perspective, this review clearly shows that a systems approach provides a useful and powerful tool to build a comprehensive framework for performance evaluation of both the overall nursing system and its subsystems. By incorporating the three functions described above, the NCPF offers an effective way to compile a complete picture of overall nursing care performance in any setting. It suggests a shift away from a narrow view of nursing performance to a broader perspective that encompasses more complex and more complete measures that take into account extended aspects of nursing activities. As such, the NCPF’s systemic perspective may help avoid many of the dysfunctional strategies that often guide performance measurement [102-105]. One such dysfunctional strategy, often described as tunnel vision, consists of concentrating on certain limited areas of performance while neglecting other important but unmeasured areas. Another such strategy is to focus on short-term issues and proximal outcomes that are more easily detectable, to the exclusion of long-term issues or distal outcomes, which may be more difficult to measure or take some time to become measurable. A third strategy is to focus on indicators that are more readily available (in most cases, structural indicators) while overlooking more complex measures. Finally, another strategy is to focus on isolated indicators without taking into account the many interactions between the different components of the system. The NCPF may help mitigate these dysfunctions. This framework offers managers appropriate guidance to devise a set of performance indicators that encompasses all functional areas of the nursing system, covers its structural features as well as processes and outcomes, considers both short-term and long-term issues, and provides a cross-functional view of the nursing system and of the interdependence among its different functions. While many aspects of nursing care may remain irreducibly invisible to policy-makers, the NCPF brings attention to a wide array of definable and measurable dimensions that can be used to assess nursing contribution.

However, while such a framework reflects the multidimensionality of nursing care performance, it poses the serious challenge of selecting a core set of indicators from among multiple options. The challenge is to find a fair balance between a narrow, easily implemented set of indicators that provides a limited picture of performance and a larger, more complex, but unwieldy set of indicators that gives a more complete picture. Some authors warn that having an unbalanced and overabundant set of performance measures can be counterproductive. Pushing staff to deliver an unreasonable number of targets may increase stress, lower morale and ultimately compromise quality and performance [105]. The NCPF may be a useful tool to address such challenges.

However this study only achieves the first requirement in the development of any performance measurement system, which is to develop a robust conceptual framework within which performance indicators can be identified and developed. The foundations provided by the NCPF would ensure that all major areas of nursing care performance are covered by any measurement system, that priorities for developing new measures or instruments can be identified, and that data collection efforts on performance are not misdirected or duplicated.

While the NCPF provides a matrix of indicators that covers the key functions of the nursing system, this framework is based on the principle that performance measurement, by its dynamic nature, must address both short-term and long-term issues and be constantly adjusted to the challenges of particular domains at particular times [106-108]. While the framework specifies what core aspects should be measured to assess nursing care performance, it allows for variations in the combinations of indicators used to measure those aspects in different contexts of care. Many jurisdictions are faced with the challenge of developing a realistic set of performance indicators with sufficient breadth and depth to capture the spectrum of nursing care. The NCPF provides a common ground from which to define performance, decide what is meaningful and important with regard to each nursing subsystem or function, devise a common and balanced set of performance indicators for a given sector of nursing care, and derive benchmarks for this sector. In short, the challenge for policy-makers is to select from among the universe of variables incorporated into this framework to develop, for the different sectors of nursing, an optimal and common portfolio of performance indicators that do not simply focus on measuring what is available or easy to measure, but are consistent with their objectives, account for their context of care and are relevant to the needs of the key stakeholders. Such selection must be informed by explicit criteria for ensuring that performance indicators exhibit the key characteristics of acceptability, feasibility, reliability, sensitivity to change and validity [109-111].

Developing a performance measurement system for nursing care is also about collecting, computing and presenting performance data for the purposes of following up, monitoring and improving organizational and clinical performance. Many of the current efforts to use performance data tend to concentrate on collecting and organizing existing administrative information and disseminating it for limited management applications [112]. To use this framework to best advantage, policy-makers and managers must invest in more creative ways of collecting, analyzing and reporting the data, and ultimately applying that information to improve nursing performance. The diverse nature of the dimensions of nursing care performance, the multiple possible uses of the performance data, and the variety of audiences involved may necessitate a wide variety of data collection methods, analytical techniques and dissemination strategies to assemble this information, make it accessible to different groups, and ensure that positive action results from it.

Despite the contributions mentioned above, some limitations remain in this study. Although we used a highly elaborated and systematic protocol to conduct the review, the approach was challenged by the broad nature of the topic being reviewed. It required a breadth of search and a labour-intensive process that allowed us to identify a rich set of performance-related variables and linkages between them. However, the number of themes and issues to cover may have limited the depth of the review on specific aspects of performance measurement in nursing care. More in-depth complementary reviews will be needed to document specific dimensions or subdimensions of this framework or to compile more thorough evidence on the linkages identified between those dimensions. While the review revealed some indicators that are more prevalent in existing conceptual models or are supported by strong research evidence, it is premature to conclude those elements are more significant than others that have been the subject of less attention or research. This is an unfinished agenda that opens the door for extensive research programs. Further studies will be needed to assess the implementation of the NCPF in different contexts of nursing care, gain further insight into the linkages hypothesized in the framework, and compile more evidence on the indicators that are more sensitive to nursing and that address the needs of the key stakeholders involved in care. Finally, another limitation may be that, while our search focused on the performance literature, we may have overlooked some key dimensions that do not relate directly to performance but may be essential to ensure a system’s performance.

## Competing interests

The authors declare they have no competing interests.

## Authors’ contributions

The study was conceived and designed by Carl-Ardy Dubois and Danielle D’Amour. All authors made a substantive contribution to the four phases of this systematic review, as described in the methods section. Carl-Ardy Dubois prepared the first draft of this manuscript. All authors contributed to revising the manuscript. All authors read and approved the final manuscript.

## Pre-publication history

The pre-publication history for this paper can be accessed here:

http://www.biomedcentral.com/1472-6955/12/7/prepub
